# The effectiveness of digital training on screening, brief interventions, and referral to treatment (SBIRT) for medical and health professionals: a systematic review

**DOI:** 10.1093/bmb/ldaf013

**Published:** 2025-09-22

**Authors:** Holly Blake, Wendy J Chaplin, Alisha Gupta, Frank Coffey

**Affiliations:** School of Health Sciences, University of Nottingham, Queen's Medical Centre, Nottingham, NG7 2HA, United Kingdom; NIHR Nottingham Biomedical Research Centre, Nottingham University Hospitals NHS Trust, Queen's Medical Centre, Nottingham, NG7 2UH, United Kingdom; School of Health Sciences, University of Nottingham, Queen's Medical Centre, Nottingham, NG7 2HA, United Kingdom; NIHR Nottingham Biomedical Research Centre, Nottingham University Hospitals NHS Trust, Queen's Medical Centre, Nottingham, NG7 2UH, United Kingdom; Population Health Sciences Institute, Newcastle University, Newcastle-upon-Tyne, NE1 7RU, United Kingdom; School of Health Sciences, University of Nottingham, Queen's Medical Centre, Nottingham, NG7 2HA, United Kingdom; DREEAM - Department of Research and Education in Urgent and Emergency Care, Nottingham University Hospitals NHS Trust, Nottingham, NG7 2UH, United Kingdom

**Keywords:** digital training, screening, brief interventions, referral to treatment, public health, systematic review

## Abstract

**Introduction:**

The effectiveness of digital SBIRT training for improving knowledge/competence and confidence for health promotion, behavioural and/or health outcomes is not established. We aimed to conduct a systematic review examining the effectiveness of digital training for medical and health professionals on screening, brief interventions, and referral to treatment (SBIRT), on knowledge of the health condition/behaviours, their treatments, and onwards referral to services, and/or changes in attitude, skills, or confidence to promote health.

**Source of data:**

MEDLINE, EMBASE, CINAHL, PsycINFO, Epistemonikos, Google Scholar, and SCOPUS. Forty-two articles with 8985 participants, published between January 2001 and April 2024, were included. There were eight randomized controlled trials. Only one study was conducted in the UK.

**Areas of agreement:**

Digital SBIRT training may increase knowledge/competence, confidence, and self-efficacy for SBIRT delivery.

Focus is primarily alcohol, tobacco, and substance use. Delivery is mostly web-based programmes, digital patient simulation, or blended learning with a face-to-face component.

**Areas of controversy:**

Comparison between studies is hampered by heterogeneity in study design, target populations, intervention design and content, comparator/control groups, and outcomes assessed.

**Growing points:**

Majority of studies were cohort educational web-based learning. Studies were mostly low quality (13/42 with low risk of bias). Outcomes were diverse and often poorly reported.

**Areas timely for developing research:**

More high-quality research is needed, including assessment of practice, behavioural, and health outcomes. A standardized approach to assuring quality of delivery and testing is required. There is scope to develop, evaluate, and implement SBIRT interventions in a broader range of health promotion areas.

## Introduction

Screening, brief intervention, and referral to treatment (SBIRT) is an evidence-based public health approach to identifying and delivering services to those with health risks. These risks relate to modifiable behaviours often associated with alcohol, tobacco, and substance misuse, physical inactivity, obesity, sexual health, and mental health conditions. SBIRT is perceived by healthcare professionals as an important approach to public health [1] and has been associated with cost savings and health benefits in some health contexts and clinical settings [2]. The practice of SBIRT aligns with national agendas for Making Every Contact Count (MECC), which advocates behaviour change through the opportunistic delivery of consistent and concise healthy lifestyle information within routine health and care interactions [3]. SBIRT, however, is not universally and routinely implemented in clinical practice [1]. There are myriad reasons, but one is the limited evidence for its effectiveness, with prior reviews often focused on specific health areas such as alcohol and substance use, and/or specific health contexts, such as emergency care, with inconsistent findings [4,5]. Another reason is that healthcare workers report a lack of training in SBIRT as a key barrier to its implementation, alongside poor knowledge, skills, or experience and low motivation, confidence, or self-efficacy for implementing SBIRT1 [6–8].

The UK Institute for Health Promotion and Education’s (IHPE) Position Statement on ‘Health Promotion’ states that ‘access to educational programmes should be made available for all those involved in providing health promotion in whatever capacity and at whatever level… this includes those who spend a small amount of time on this activity as well as health promotion specialists’ [9]. To align with such guidance, SBIRT training is needed for health and care professionals to equip them to implement SBIRT in clinical practice and support the improvement of population health. In recent years, SBIRT training has been increasingly (albeit inconsistently) delivered to qualified medical and health professionals as continuing professional development and embedded within graduate training curricula for medical and health trainees across a range of professions, including physicians, physician assistants, nurses, physical therapists, occupational therapists, and psychologists. Training is provided using a range of delivery modalities, including face-to-face approaches, blended learning (which combined traditional face-to-face instruction with technology-mediated online instruction), or solely via digital platforms such as web or app-based programmes. The COVID-19 pandemic accelerated ‘digital’ approaches to learning worldwide, which has continued to proliferate. Digital learning is defined as ‘learning that is facilitated, enabled or mediated using electronic technology for the explicit purpose of training, learning or development’ [10]. Digital training has broad benefits for both health educators and learners. It is low cost compared to face-to-face delivery, has potential for wide geographical reach, offers consistency and standardization of materials, and is flexible and convenient for users who have greater personal control over how and where materials are accessed [11,12]. As with many areas of medical and healthcare training, digital training for SBIRT is growing in popularity [1], but its effectiveness is not known. Prior systematic reviews related to SBIRT focus on individual health behaviours (e.g. addictive behaviours [13], substance use [5,14,15], alcohol [16]), specific target populations for SBIRT practice (e.g. adolescents [17]), or specific training recipients (e.g. healthcare trainees [18]). To our knowledge, no prior review has been conducted that focuses on digital delivery of SBIRT training, in any health or behavioural area, with any population of training recipients, or any target audience for SBIRT implementation.

### Study aim

To conduct a systematic review to examine the effectiveness of digital training for medical and health professionals on SBIRT, on knowledge of the health condition/behaviours, their treatments, and onwards referral to services, and/or changes in attitude, skills, or confidence to promote health.

## Methods

This systematic review was pre-registered with PROSPERO on 22 April 2024 (CRD42024526403). This review was conducted according to the Preferred Reporting Items for Systematic Reviews and Meta-Analyses: the 2020 PRISMA statement [19].

### Eligibility criteria

We included all original studies consisting of randomized control trials (RCTs) and quasi-experimental studies, that included an element of digital training and were published after 2000—when the term ‘eHealth’ first emerged. The included studies were without geographical limitation but were restricted to the English language. Participants were medical and healthcare professionals in any health or social care settings. Digital training was a constituent of the intervention or training, with delivery modality including (but not restricted to) web-based or app-based tools, with delivery undertaken as part of medical education or healthcare training. This included blended learning which combined digital training elements with additional classroom education but excluded solely classroom or other face-to-face delivery. Comparator included paper-based information, classroom-only, or no-intervention controls. Outcomes were measured as changes in the effect of the intervention, on knowledge of the health condition/behaviours, their treatments, and onwards referral to services, and/or changes in attitude, skills, or confidence to promote health. Reviews, opinions, letters, conference proceedings, and unpublished literature were not considered.

### Search strategy

The following databases were searched electronically: MEDLINE, EMBASE, CINAHL, PsycINFO, Epistemonikos, Google Scholar, and SCOPUS. Searches were conducted in April 2024. The search strategy will combine the following concepts and study-type filters: screening, brief interventions, and referral to treatment (SBIRT), digital, computer-based, internet-based, or mobile applications. The MEDLINE search can be found in Supplementary file S1.

### Study selection

Two authors (W.J.C., A.G.) were involved in study selection. Records were managed through Covidence systematic review software (Veritas Health Innovation, Melbourne, Australia). An initial screening of titles and abstracts of studies retrieved was conducted (W.J.C.), to identify studies that met the study inclusion criteria outlined above. A second reviewer (A.G.) independently screened 20% of titles and abstracts. Full text was obtained for abstracts with insufficient information or in a situation of disagreement. A study was included when two reviewers (W.J.C., A.G.) independently assessed it as satisfying the inclusion criteria from the full text. Inter-rater agreement using Cohen’s Kappa was 80.05%. Any disagreements were fully resolved through discussion.

### Data extraction

Two authors (W.J.C., A.G.) were involved in data extraction which was undertaken using Excel. Data extraction was independently performed on all included articles by both authors. The following data were extracted (Supplementary file S2): author and year, name of the journal, study design, inclusion/exclusion criteria, number of participants, participant characteristics (age, gender ratio), type of intervention, results, and outcome measures. Any disagreements were fully resolved through discussion.

### Study risk of bias assessment

All included studies were independently assessed for risk of bias by two reviewers (W.J.C., A.G.) with an initial agreement of 80.05%. Disagreements were resolved by discussion between the reviewers to reach consensus. The JBI Critical Appraisal Tool for RCTs [14] and Quasi-Experimental Studies [15] were used as appropriate (Supplementary file S2).

### Data synthesis

Synthesis involved a narrative description of study designs and settings, target population characteristics, training characteristics (such as topic area and mechanisms for training delivery), and type of outcomes, summarized in a table. Outcomes of interest included but were not limited to: knowledge of the health condition/behaviours, their treatments, and the referral services; changes in attitude, skills, or confidence to promote health; and SBIRT implementation or delivery. Studies were grouped according to intervention components, study design (RCT, observational design, etc.), setting, intervention target such as alcohol or substance(s), target populations, and outcomes. No meta-analysis was planned, due to high heterogeneity in study methodology and outcome presentation in the field of SBIRT using digital applications. Findings were synthesized through interpretation and critical discussion of findings, including variability in outcomes, interventions, study designs, settings, and biases.

## Search results

### Study selection

**Figure 1:**
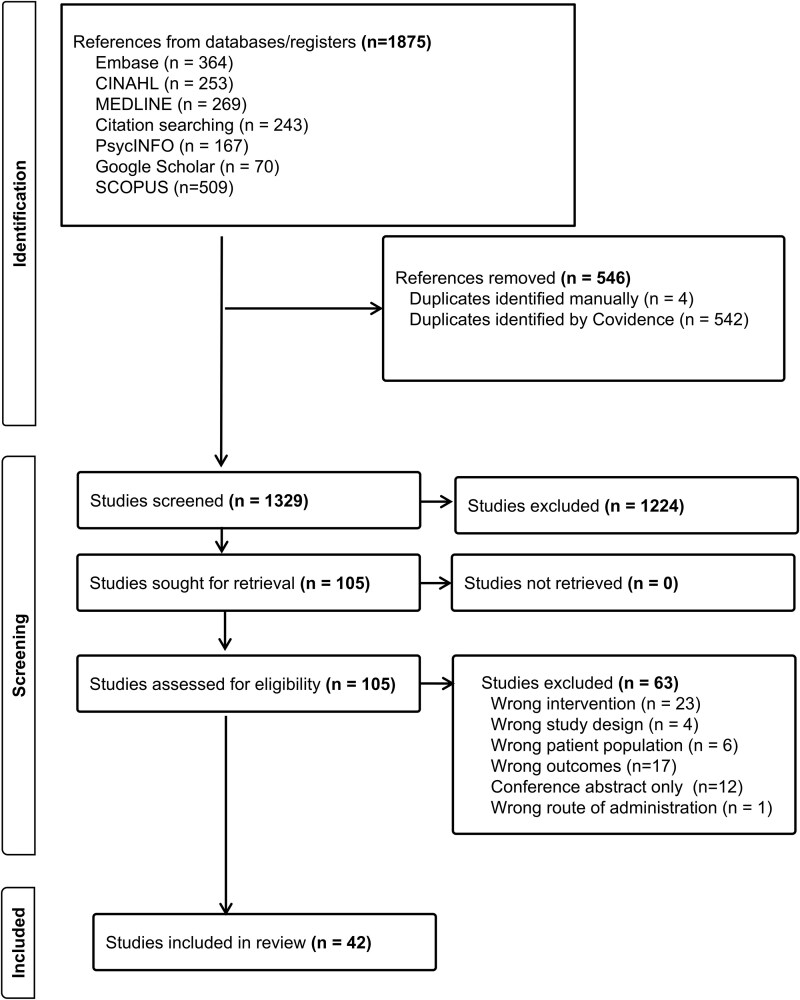
PRISMA flow diagram.

The search identified 1875 records and 105 full articles that met the inclusion criteria for further examination. Sixty-three articles were excluded. Forty-two included articles were published between 2007 and 2024 (33 of which were published 2015 onwards). Figure 1 provides a flowchart of the literature search, including reasons for exclusion.

### Study designs and settings

The 42 studies included eight RCTs [20–27] and 34 quasi-experimental designs. One study was conducted in the UK [28], 2 in Australia [29,30], and the remaining 39 studies were conducted in the USA [20,21,23–26,31–60]. Training settings included 1 study based in urgent and emergency care [32]; 3 in hospital in-patient care [25,34,38]; 1 in primary care (a general practice: GP) [30]; 17 were university student cohorts from medical, health, and social care disciplines [21,23,24,29,31,36,37,39–48,52–55,57–59,61]; and 10 were in other organizations employing healthcare professionals (e.g. counselling services or hospitals, through distance learning, virtual, or online courses) [20,22,26,28,33,35,49–51,56].

### Target population characteristics

A total of 8985 participants were included in the 42 studies. Training recipients were urgent and emergency care staff (*n* = 402), GPs (*n* = 18), other healthcare professionals (e.g. counsellors, physiotherapists, dieticians), nurses (working in in-patient settings) (*n* = 286), or university students (*n* = 5955). Approximately 5782 participants were female. However, not all studies declared their sample demographics.

### Intervention characteristics

Nine studies focused solely on alcohol use/misuse [22,27,32–34,42,57–59]. Four studies focused on drug or substance use/misuse [35,36,46,47]. Twenty studies combined their focus on both drug and alcohol use/misuse [21,24,31,37–41,43–45,48–55,60]. Four studies focused on alcohol, tobacco, and other drugs (ATODS) [23,26,30,61]. Four studies focused on mental health and health behaviours [20,25,28,29], and one study focused on vitamin D deficiency [56]. Twenty-one studies used web-based training [23,25,27,32–34,37,38,44–47,50,51,54–58]. Eight studies included a blended element of web-based learning alongside classroom learning or role-play [28–31,35,39,43,48]. Three included web-based training with a digital simulated patient training element [40–42]. Nine studies focused on digital simulated patient training [20,22,24,36,49,52,53,60,61]. One study employed a mobile application [21].

### Type of outcomes

There was a wide range of outcomes. To permit comparison between heterogeneous studies, outcomes have been categorized into knowledge, competence, confidence, and a summary of ‘other’ findings (Table 1). Twenty-two studies measured change in knowledge [20,23,25,31,35–44,47,49,51,54,56–58,61], 6 studies measured perceived competence [28,31,41,46,49,50], and 15 measured confidence [21,23,24,28,30,32,38–41,54,56,59–61]. Other outcomes included screening behaviour (in clinical practice and/or examination as part of training) [20,26,27], scenario testing [22], patient management and patient engagement [24], readiness (for screening or interprofessional education) [23,39,49,50], empathy and adherence [23], responsibility, and/or utilization and barriers [32,59], self-efficacy [33,36,43,47,57], satisfaction [47], role adequacy and performance [34], self-perceived ability [55], attitudes (e.g. to SBIRT recipients, to SBIRT practices and/or to own skills or knowledge) [21,24,40,41,44,45,48,49,51,54] [58], subjective norms [21], motivation enhancement [21], behavioural intentions [21,28,42], role support [44], and motivational interviewing skills [29,52,53].

**Table 1:** Characteristics of included studies and outcomes.

**Lead author, year of publication** **[citation]**	**Study** **design**	**Country**	**Funding**	**SBIRT target**	**Setting**	**Participants** N (% female**)**	**HCP ROLE**	**Digital element**	**Knowledge** (SD)	**Competence** (SD)	**Confidence** (SD)	**Other** (SD)
Acquavita *et al.*, 2021 [31]	Quasi	USA	SAMHSA	Drug and alcohol	University student cohort	216 (69.7)	Medical, graduate pharmacy, undergraduate senior/master-level social work/undergraduate (junior/senior)/graduate nursing students	Web-based training and blended (50 h)	**Pre 12.25 (3.17**) **Post 15.55 (1.65)**	**Pre 29.81 (6.71** **Post 18.41 (4.80)** **Lower Likert-type scores signified a higher level of perceived competence**		
Albright *et al.*, 2018 [20]	RCT treatment and waitlist control	USA	None	Trauma-related mental health disorders that were defined as alcohol and substance abuse, depression, generalized anxiety disorder, and posttraumatic stress disorder	Practicing primary HCPS	227 (81.9)	Primary HCP	Virtual patient simulation	**Pre 2.82 (0.79)** **Post** **3.40 (0.89), *P* < .001** **Cohens d = 0.61**			**Screening behaviour** **Treatment group 3.27 (0.74)** **Control group** **2.90 (0.87) *P* < .01 Cohen’s *D* = 0.45**
Bernstein *et al.*, 2007 [32]	Quasi	USA	NIAAA	Alcohol	14 Emergency Dept sites associated with teaching hospitals	402 (48)	ED physicians, nurses, nurse practitioners, physicians’ assistants, social workers and Emergency Medical Technicians	Web-based training			**Pre 3.75** **Post 4.15, *P* < .001**	Responsibility, utilization, barriers
Bray *et al.*, 2009 [33]	Quasi	USA	NIAAA	Alcohol	Employee Assistance Programs Counsellors in 26 offices	74 (68)	‘Employee Assistance Programs EAP counsellors Credentials. Certified Employee Assistance Professional. Licensed Chemical Dependency Counsellors. Licensed Clinical Social Workers. Licensed Marriage and Family Therapists. Licensed Professional Counsellors.	Web-based training				Self-efficacy Pre 19.37 Post 20.21
Broyles *et al.*, 2013 [34]	three-phase pilot study Quasi	USA	Veterans Affairs Quality Enhancement Research Initiative Program	Alcohol	Nurses in a hospital for Veterans	78 (93)	Nurses	Web-based training				After training, increase in role adequacy, increased performance and competence for more SBIRT tasks
Bull & Dale, 2021 [28]	Quasi	UK	None	Health behaviours	NHS employees	177 (88)	Health and social care practitioners working in North East Scotland	Web-based training and blended		**Information about health consequences 7.48 (1.19)** **Pros and cons 7.96 (1.17)** **action planning 7.87 (1.37)** **self-monitoring beh 7.93 (1.35)** **prompts and cues7.89 (1.41)**	**Information about health consequences 7.54 (1.29)** **Pros and cons 8.03 (1.26)** **action planning 7.94 (1.36)** **self-monitoring beh 8.05 (1.4)** **prompts and cues 8.04 (1.39)**	Intention
Cambron *et al.*, 2023 [35]	Quasi	USA	Health Resources and Services Administration of the US Department of Health and Human Services under M01HP31280 and the Behavioural Health Workforce Education and Training Program.	Drugs in prenatal	Substance use counsellors’ online course	100 (62)	Paraprofessional substance use disorder counsellor students enrolled in an in-person undergraduate SUDC certificate program at a public university	Web-based training and blended	**Pre 3.3 (1.2)** **Post 3.9 (1.2) *P* = .003** **Effect size 76%**			
Cordes *et al.*, 2022 [36]	Quasi 2 group	USA	SAMHSA	Drugs	University student cohort	293 (78.5)	Undergrad (180) grad beh health (63) grad medical (50)	Patient simulation	**Pre 0.71 (0.13)** **Post 0.78 (0.14), *P* < .001**			Self-efficacy Pre-screening 37.46 (31.95) In BI 35.97 (29.07) In RT 40.88 (34.8) Post-screening 70.19 (23.22) In BI (65.31 (22.95) in RT 74.59 (24.10)
Curtis *et al.*, 2022 [21]	RCT	USA	SAMHSA	Drugs and alcohol	University student cohort	131(74.5)	Multi-disciplinary health professionals’ trainees	Mobile app			Pre 6.33 (1.71) Post 6.73 (1.46), ns	Attitudes, subjective norms, behaviour intention Utilities: Theory of Planned Behaviour
Fleming *et al.*, 2009 [22]	RCT	USA	NIAAA	Alcohol	Organisations employing healthcare professionals	731	HCPs	Patient simulation				**Scenario testing** **Pre Screening** **53.24 (16.09)** **Post Screening 67.67 (12.42) *P* < .01** **Pre BI** **52.55 (13.86)** **Post BI 58.37 (15.89) *P* = .05** Pre-referral 42.94 (14.28) Post-referral 66.05 (14.02) ns
Gainey *et al.*, 2022 [61]	Quasi	USA	SAMHSA	Illicit substance and misuse of prescription drugs, alcohol, and tobacco.	University student cohort	1229 (64.4)	Undergraduate and graduate nursing students, medical students.	Patient simulation	**Pre 69.6 (13.2)** **Post 88.1.(16.7) d = 1.2 effect, large**		**Pre 2.8 (0.9)** **Post 2.1 (0.6) d = 0.9 effect, large**	
Giudice *et al.*, 2015 [23]	RCT	USA	SAMHSA	Alcohol and smoking cigarettes, undesirable adolescent behaviours (paeds) focused SBIRTS	University student cohort	40	Paediatric residents	Web-based training	**Pre 0.29 (0.17)** **Post 0.41 (0.18)**		**Screen** Pre 5.53 (2.42) **post 8.20 (1.21)** **BI** Pre 5.4 (1.99) **Post 8.18 (1.06)**	Behaviours, readiness, empathy adherence
Gonzalez *et al.*, 2020 [38]	Quasi	USA	Manuscript funding	Drugs and alcohol	Hospital in-patient care	40 (80)	Nurses	Web-based training	**Pre 51.3 (16.4)** **Post 79.8 (15.9) *P* < .001**		Mean 6/7 ‘agree’ alcohol, and 5/7 ‘somewhat agree’ drugs	
Gonzalez *et al.*, 2021 [37]	2 group both online	USA	SAMHSA	Drugs and alcohol	University student cohort	169 (80)	Nursing students and registered nurses	Web-based training	**Pre 79.33 (17.31)** **Post 80.88 (15.77)**			
Habib *et al.*, 2019 [39]	Quasi	USA	SAMHSA	Drugs and alcohol	Hybrid University/HCP training	497 (86)	Social work (master’ s-level), nursing (master’s and nurse practitioner), and psychology (master’s and mental health counselling) graduate-level students received training in SBIRT, as well as field professionals.	Web-based training and blended	Pre 4.68 (1.57) **Post 6.13 (1.87) *P* < .001**		Pre 6.66 (3.2) Post 7.23 (1.8) ns	Readiness to screen
Kelly *et al.*, 2018 [40]	Nonrandomized 2 group	USA	SAMHSA	Drugs and alcohol	University student cohort	63 (94)	Adult/geriatric and family Master of Science in Nursing nurse practitioner students	Web-based training and simulated patient	**Pre 9.11** **Post 10.89, *P* < .001**		**Pre 1.68** **Post 2.18 *P* = .049**	AAPPQ DDPPQ
Knopf-Amelung *et al.*, 2018 [41]	Quasi	USA	SAMHSA	Drugs and alcohol	University student cohort using blended approaches comparison of 3 approaches	256 (81.5)	BSN Nurses	Web-based training and simulated patient	**Pre 8.1 (1.7)** **Post 10.3 (1.9)**	BI Competence post-training 8.9 (0.8) (post only)	**Pre 1.4 (0.7)** **Post 2.1 (0.7)**	AAPPQ DDPPQ MI style post only 3.7 (1.4)
Lee *et al.*, 2008 [42]	Nonrandomized 2 group	USA	Health Research Award	Alcohol	University student cohort comparison of lecture vs web module	163	First-year medical students	Web-based training and simulated patient	**Pre 72** **Post 86 *P* < .005**			**behaviour intervention skills total (1–13)** **9, *P* < .02 effect 0.38**
Martin *et al.*, 2020 [43]	Quasi	USA	SAMHSA	Prescription and illicit drugs and alcohol	University student cohort	27 (80)	Psychology trainees	Web-based training and blended	**Pre 15.41 (1.37)** **Post m = 16.76 (2.05), *P* = .015 partition n2 = 0.232**			Self-efficacy Post (M = 7.69, 95% CI [0.42, 14.96], *P* =.033)
Mitchell *et al.*, 2017 [44]	Quasi	USA	SAMHSA	Drug and alcohol	University student cohort	80 (53)	Medical residents	Web-based training	**Role adequacy** **Pre 33.89 (5.04)** **Post 38.10 (6.60) *P* < .001**			**AAPPQ** **Role support** **Pre 15.54 (4.00)** **Post 17.08 (3.22) *P* = .005**
O’Brien *et al.*, 2019 [24]	RCT	USA	SAMHSA	Drugs and alcohol	University student cohort	308 (85)	Social work and nursing students	Patient simulation			Pre 4.15 (1.43) Post 3.02 (0.92) NS Lower is an improvement	Importance, attitudes
Osborne *et al.*, 2016 [45]	Quasi	USA	SAMHSA	Drugs and alcohol	University student cohort	76 (87)	Social work students	Web-based training				Attitude, knowledge and skills 8/14 questions were significantly different post-intervention. Lower scores = higher agreement.
Oster *et al.*, 2022 [29]	Feasibility	Australia	Flinders Foundation Health Seed Grant	Health behaviours with drug and alcohol	University student cohort	41 (90.9)	Postgraduate students in various health professional courses, including Chronic Condition Management, Cognitive Behavioural Therapy, Physiotherapy, Occupational Therapy, Nutrition and Dietetics, and Nursing.	Web-based training and blended				**Relational MITI score** **Pre (2.78 (0.78)** **Post 3.23 (0.70), *P* = .016, medium effect 0.59** **Technical MITI** **Pre 2.9 (0.72)** **Post 3.42 (0.61), *P* = .006, large effect 0.8** **PMAAQ** **Pre 2.44 (0.55)** **Post 2.18(0.41), *P* = .025, medium effect 0.54**
Petrides *et al.*, 2024 [46]	Quasi	USA	SAMHSA	Substance	Osteopathic medical school and primary care residents and students	144 (81)	Third & fourth year osteopathic medical students and primary care residents	Web-based training		**Increase 6.5%, *P* < .01**		
Pickard *et al.*, 2024 [47]	Quasi	USA	SAMHSA	Substance	Two University student cohorts and HCPS completed an online course	1200 (85.9)	Social work students	Web-based training	**Pre 13.29 (3.24)** **Post 16.25 (2.8), *P* < .001**			Efficacy Satisfaction
Pringle *et al.*, 2017 [48]	Quasi	USA	SAMHSA	Drugs and alcohol	University comparison of 7 statewide SBIRT medical residency programs	365 (48)	Residency training programme	Web-based training and blended	**Pre 621** **Post 832, *P* < .001**			AAPPQ **DDPPQ** **Pre avg 22.35 (6.02)** **Post avg 21.04 (6.29), *P* = .003**
Puskar *et al.*, 2016 [50]	Quasi	USA	Division of Nursing (DN), Bureau of Health Professions (BHPr), Health Resources and Services Administration (HRSA), Department of Health and Human Services (DHHS)	Drugs and alcohol	HCPS in 3 rural areas	101 (85.2)	Healthcare professionals (nurses, behavioural health counsellors, and public health workers)	Web-based training		**(*F* = 4.07, *P* = .02), with an increase of 0.14 between pre- and post-training,**		**IEPS perception of actual cooperation** **(F = 2.21, *P* = .11)** **IEPS Understanding other value** **(F = 6.21, P < .01), with an increase of 0.16 between pre- and post-training, and an increase of 0.39**
Puskar *et al.*, 2016 [49]	Quasi	USA	DN, BHPr, HRSA (DHHS)	Drugs and alcohol	Compares nurses with behavioural health professionals in in 3 rural areas	98 (82)	62 Registered nurses and 36 Behavioural Health Professionals	Patient simulation	**Alcohol** **Pre 3.45 (0.72)** **Post 3.72 (0.65), *P* = .05** **Drug** **Pre 3.31 (0.66)** **Post 3.58 (0.60), *P* = .01**	**Pre 4.92 (0.74)** **Post 5.14 (0.62), *P* = .05**		AAPPQ DDPPQ IEPS
Puskar *et al.*, 2016 [51]	Quasi	USA	DN, BHPr, HRSA (DHHS)	Drugs and alcohol	HCPS in 3 rural states	106 (84.9)	Healthcare professionals (nurses, behavioural health counsellors, and public health workers) from rural areas in Pennsylvania, Ohio, and West Virginia.	Web-based training	**Alcohol** **Pre 3.6 (0.81)** **Post 3.84 (0.65), *P* = .01** **Drug** **Pre 3.47 (0.77)** **Post 3.72 (0.67), *P* = .02**			AAPPQ DDPPQ
Putney *et al.*, 2019 [53]	Quasi	USA	SAMHSA	Drugs and alcohol	University student cohort online patient simulation to boost clinical skills with representative populations	19 (79)	Social work students	Patient simulation				MI (total) Pre 18.05 (5.91) Post 34.74 (4.51) *P* <.001 MI (mechs) Pre 13.89 (5.18) Post 21.74 (3.07), *P* <.001
Putney *et al.*, 2021 [52]	Quasi, pilot	USA	SAMHSA	Drugs and alcohol	University student cohort using a blended approach	54 (82)	Masters social work students	Patient simulation				**Alcohol, Drugs and Social Work Practice Total** **Pre 62.52 (16.48)** **Post 75.89 (13.5), *P* < .001** **Screening** **Pre 9.49 (4.64)** **Post 16.77 (5.08), *P* < .001** **BI** **Pre 10.24 (4.82)** **Post 14.55 (3.4), *P* < .001** Patient Management Pre 42.79 (11.61) Post 44.64 (11.24), ns **MI (total)** **Pre 42.79 (11.61)** **Post 74.81 (12.77), *P* < .001** **MI Mechs** **Pre 23.29 (7.91)** **Post 40.38 (7.61), *P* < .001** **Patient engagement** **Pre 26.05 (7.47)** **Post 30.0 (0), *P* = .25**
Rawlings *et al.*, 2019 [54]	Quasi	USA	SAMHSA	Drugs and alcohol	Three faith-based University student cohort for allied healthcare programs	251 (88.85)	Social work students UG and PG	Web-based training	Pre 2.37 se 0.03 Post 4.03 se 0.04		Pre 3.53 SE 0.05 Post 4.15 SE 0.04	Faith Attitudes, Knowledge and Skills
Rittle *et al.*, 2019 [55]	Quasi	USA	SAMHSA	Drugs and alcohol	University student cohort partnership between 2 universities and a federal agency	128	Training of Health Champions within the Physician Assistant, Occupational Therapy, Physical Therapy, Nursing, and Counselling Psychology programs	Web-based training				Self-perceived abilities
Ruzek *et al.*, 2014 [25]	RCT	USA	US Army Medical Research and Materiel Command	PTSD	Veterans Health Administration clinicians working with veterans in a clinic providing PTSD Treatment	168 (69.6)	Full-time VHA mental health clinicians experienced with veterans with PTSD	Web-based training	CBT knowledge Pre 7.59 (1.89) and 7.95 (1.97) Post 8.85 (1.91) and 9.67 (1.65) P,0.001			Motivation enhancement Pre 0.57 (0.28) and 0.58 (0.33) Post 0.69 (0.34) and 0.93 (0.37), *P* <.001 Goal setting Pre 0.84 (0.32) and 0.81 (0.36) Post 0.88 (0.46) and 0.99 (0.35), *P* =.007 Behaviour task assessment Pre 0.62 (0.34) and 0.65 (0.32) Post 0.79 (0.41) and 0.92 (0.44), *P* <.001 SR skills implementation Pre 3.58 (0.72) and 3.50 (0.72) Post 3.95 (0.66) and 0.3.73 (0.79), *P* <.001
Sanford *et al.*, 2023 [56]	Quasi	USA	None	Vitamin D deficiency	Increasing HCP awareness of vitamin D deficiency via an online toolkit	119	102 (86%) nurses, dieticians, 16 (13%)	Web-based training	Pre 31% Post 65%, *P* <.0001		Pre 2.0 Post 3.3, *P* <.0001 Scale 1–5 Paired *t*-test	
Stevens *et al.*, 2024 [30]	Mixed methods pilot	Australia	COORDINARE—Southeastern NSWPHN through the Australian Government’s PHN program	Alcohol, tobacco, and other drugs (ATODS)	General practice in rural Australia	18 (45)	GPs	Web-based training and blended			Confidence to screen pre 59.7 (17.0) post 67.5 (11.5) ns Confidence BI Pre 56.7 (22.1) Post 71 (9.1), ns	
Stoner *et al.*, 2014 [26]	RCT	USA	National Institute on Drug Abuse, National Institutes of Health, Department of Health and Human Services to Talaria	Alcohol, tobacco, and other drugs (ATODS)	National RCT comparing online training with reading materials for HCPS	161 (67)	Physicians, nurse practitioners, and physician assistants	Web-based training				**Counselling–drink** **Pre 1.01 (0.1)** **Post 1.47 (0.06), *P* < .001** **Counselling–drug abuse** **Pre 0.49 (0.10)** **Post 1.17 (0.06), *P* < .001** **Counselling—illegal drugs** **Pre 0.24 (0.13)** **Post 0.93 (0.08), *P* < .001** **Screening alcohol** **Pre 0.11 (0.12)** **Post 0.87 (0.1), *P* < .001** **Screening Drug misuse** **Pre 3.3 (0.2)** **Post 4.6 (0.1), *P* < .001** **Screening illicit substances** **Pre 2.2 (0.2)** **Post 4.2 (0.1), *P* < .001** **Screening tobacco** **Pre 3.1 (0.2)** **Post 4.3 (0.1), *P* = 0.005** **Behaviours screening** **Pre 4.6 (0.1)** **Post 4.9 (0.1), *P* < .001** **Referral to treatment** **Pre 3.2 (0.2)** **Post 4.5 (0.1), *P* < .001**
Tanner *et al.*, 2012 [57]	Quasi	USA	NIAAA	Alcohol	University student cohort Real-world effectiveness trial	70	Medical students and nursing students	Web-based training	Medical Students Pre 68% Post 79%, *P* =.02 Nursing Pre 65% Post 69% *P* =.17			Self-efficacy screening Pre 3% (both groups) Post med students3.9% Post 3.7% Self-efficacy in BI Pre 3% (both groups) Post med students3.9% nurses 3.7% Patient history post only Med Students 90% Nurses 71% Differential diagnosis Med Students 80% Nurses 67%
Tenkku Lepper *et al.*, 2019 [58]	Quasi	USA	SAMHSA	Alcohol	University student cohort Development and piloting of online SBIRT training in 5 academic settings	482 (70.7)	Physician assistant students	Web-based training	**Pre 5.06 (0.61)** **Post 5.73 (0.56) *P* < .001**			**Attitude, knowledge and skills 12/14 questions were significantly different post-intervention.**
Truncali *et al.*, 2011 [27]	RCT	USA	Health Resources and Services Administration, Academic Administrative Unit grant	Alcohol	University student cohort who either completed an online module or a face-to-face lecture	141	First-year medical students	Web-based training	**Pre 45 (19)** **Post 73 (16), *P* < .001**			**Web students outperformed their peers on the OSCE in both alcohol-specific tasks (54% vs.41% items well done, P = 0.021) and general communication (65%vs.51% items well done, P = 0.004)**
Wacker *et al.*, 2023 [59]	Quasi 3 group	USA	SAMHSA	Alcohol	University student cohort in different learning environments	184 (97.2)	Master’s social work students	Web-based training (4 h course)			**Pre 3.50 (0.97)** **Post 3.98 (0.66)**	Responsibility Practice
Wood *et al.*, 2022 [60]	Quasi	USA	None	Drugs and alcohol	University student cohort	40 (82.5)	Full-time Master of Social Work (MSW) students attending a private, mid-sized intermountain university.	Patient simulation			**Pre 23.75 (6.43)** **Post 39.88 (5.28), *P* = .03**	

Abbreviations: RCT, randomized controlled trial; SAMHSA, Substance Abuse and Mental Health Service Administration; NIAAA, US National Institute of Alcohol Abuse and Alcoholism; DN, Division of Nursing; BHPr, Bureau of Health Professions; HRSA, Health Resources and Services Administration; DHHS, Department of Health and Human Services; PTSD, posttraumatic stress disorder; DDPPQ, Drug and Drug Problem Perception Questionnaire; AAPPQ, Alcohol and Alcohol Problem Perception Questionnaire; PMAAQ, Preventative Medicine Attitudes and Activities Questionnaire; IEPS, Interprofessional Education Perception Scale; OSCE, Objective Structured Clinical Examination; MI, Motivational Interviewing; BI, brief intervention; RT, referral to treatment; bold = statistically significant results.

Most studies concluded that digital SBIRT training led to improved outcomes, in particular increases in knowledge, attitude, skills, and/or confidence to promote health. However, studies were limited in scope, and outcomes were inconsistently measured and reported. Actual changes in practice (screening or brief interventions, BIs), treatments, onward referral to services, and health or clinical outcomes were rarely, if ever, reported.

### Results of risk of bias assessment

In common with many behavioural interventions, blinding was not included in many studies and the design did not permit comparison between groups. Thirteen studies had a low risk of bias [25,34–37,39–43,57,60,61]. Further details can be found in Supplementary file S2 (Tables C and D: RCT and quasi-experimental designs, respectively).

## Discussion

### Principal findings

This is the first systematic review to examine effectiveness of digital SBIRT training for improving knowledge/competence and confidence for health promotion, behavioural, and/or health outcomes. The evidence suggests that digital SBIRT training can increase knowledge, competence, confidence, and self-efficacy for SBIRT delivery in medical and health professionals and trainees. Digital SBIRT therefore has value in supporting the health and care workforce to contribute to national and international public health agendas [3,9]. Within health and social care, across a range of diverse settings, there is a wealth of ‘teachable moments’ providing opportunity for SBIRT. Prior research advocates that health professionals have unmet needs for training in SBIRT to increase their knowledge, skills, and confidence to engage in this health promotion effort [1,6–8]. The next step was therefore to examine the effectiveness of SBIRT, although prior systematic reviews have had a limited focus in terms of target populations and specific health areas [5,13–17]. There were also no existing systematic reviews that examined interventions delivered through digital platforms, although these are rapidly gaining in popularity without evidence of their effectiveness [1]. Overall, our review demonstrates that the provision of SBIRT training through digital approaches is a viable route to SBIRT delivery. We found that digital SBIRT training can promote positive attitudes towards SBIRT and provide recipients with the knowledge, skills, and confidence to engage in SBIRT practice—which could help to capitalize on these ‘teachable moments’ and support public health agendas [3,9].

The conclusions of this review are limited by the heterogeneity and quality of the included studies. Despite broadly positive findings, most of the studies in this review were of low quality; only 13 of 42 included studies were classified as having low risk of bias. Studies were predominantly quasi-experimental designs, some measuring outcomes before and after while others measured postoutcomes only. There were very few randomized controlled trials. Although there are SBIRT interventions emerging in the UK for which effectiveness has yet to be tested [62], the vast majority of included studies were conducted in the USA. Many of these US studies were funded by the same educational grants funding stream. These studies are likely to have a primary focus on ‘educational innovation’ rather than research per se. This may partially explain the high risk of bias in study designs, the lack of consistency in methodological approach and assessment of outcomes, and poor reporting of methods and outcomes. Across the 42 studies, there was significant heterogeneity in study design, target populations, intervention design and content, comparator/control groups, and outcomes assessed. Many of the included papers focused solely on one element of SBIRT—for example, the delivery of a BI in isolation, without the screening assessment or referral to treatment. Therefore, in many studies, only part of SBIRT (as defined in our review) was included in either the intervention and/or the measurement of outcomes. In this review we reported ‘other outcomes’ that were diverse and often reported by a single study leading to limited confidence in effects.

Many of the studies targeted university students, with some SBIRT interventions being delivered to healthcare professionals, including those working in urgent and emergency care settings. Using diverse and primarily self-reported outcomes (e.g. ‘perceived changes’), most studies examined knowledge and/or confidence for SBIRT, some measured attitudes, skills, and/or competence, but very few studies included any objective assessment of SBIRT practices. Those that did used digital patient simulation, which provided unique experiences (e.g. exposure to different ethnicities and cultures) and often had positive outcomes. However, no studies examined training recipients’ SBIRT practices over time or in different practice settings or contexts. Therefore, it is not possible to conclude from this review (i) whether, and how, SBIRT training is being implemented in practice and by whom; (ii) whether training recipients from different demographic or occupational groups engage with the training differently; (iii) whether SBIRT training has any lasting impacts on knowledge, confidence, or health promotion practices; or (iv) whether SBIRT training impacts on health or clinical outcomes for healthcare service users.

### Limitations of the review

We only included articles published in English, and, due to time constraints, we checked the references lists of included studies (backward reference list checking) but did not check studies that cited the included studies (forward reference list checking). Therefore, it is possible some articles could have been missed. It is possible that further evidence of changes in SBIRT practice could have been reported in other types of paper that would not have been picked up by our searches, e.g. process evaluation papers published separately to RCTs.

### Practical and research implications

The key implication from this review is that delivery of SBIRT through digital approaches could help to address unmet training needs for healthcare professionals and trainees. Digital modalities for SBIRT training have practical value since they benefit from flexibility, wide reach, and scalability [10] and could therefore make a significant contribution to supporting the healthcare workforce (and future generations) in implementing national and international public health strategies. Our review shows that most digital SBIRT training initiatives focus on alcohol and substance use, with some interventions focused on mental health trauma associated with addiction. Due to a lack of published evidence, we cannot draw any conclusions about the value of digital SBIRT in other health areas. This highlights a research gap; there is clear scope to develop and implement training in other areas of health promotion, such as mental wellbeing and stress management, obesity, weight management, physical activity, gambling addiction, or self-care and medicines adherence.

In terms of the delivery platform, most studies in this review used web-based training, digital patient simulation, or a blended approach, with one study including a mobile app. With rapidly evolving technologies, digital SBIRT training in the future could potentially use a broader range of technologies, including mobile apps, gamification, artificial intelligence (AI)-powered platforms, augmented reality, and virtual reality. These technologies, for SBIRT training, need to be developed, and tested for their feasibility, acceptability, and effectiveness.

Finally, there is a need for more high-quality research on digital SBIRT training to draw firm conclusions about the effectiveness of digital SBIRT training in the short, medium, and long term and to be able to ascertain which components of digital SBIRT training are more, or less, effective. Although prior research has developed a psychometrically reliable coding system for evaluating SBIRT interactions this is not focused on digital SBIRT, relates only to substance use [63], and, as evident from this review, has not been widely adopted in research. There is a lack of standardized approaches for other health areas. Therefore, future research should develop a standardized approach to assuring the quality of delivery and evaluation of digital SBIRT training.

## Supplementary Material

Supplementary_file_S1_ldaf013

Supplementary_file_S2_ldaf013

## Data Availability

No new data were generated or analysed in support of this review.
