# Supplementary material for: The effectiveness of digital training on screening, brief interventions, and referral to treatment (SBIRT) for medical and health professionals: a systematic review
Source: Br Med Bull. 2025 Sep 22;156(1):ldaf013. doi: 10.1093/bmb/ldaf013 (PMC12452273; doi:10.1093/bmb/ldaf013)
Supplement: Supplementary_file_S1_ldaf013 [file supplementary_file_s1_ldaf013.docx]

# **Supplementary file S1:** MEDLINE (OVID) Search strategy

Title: The effectiveness of digital training on brief interventions for health promotion for medical and health professionals.

- SCREENING, BRIEF INTERVENTION REFERRAL TO TREATMENT (SBIRT)

1. (SBIRT or e-SBIRT or e-SBI or eSBIRT or eSBI).mp.
2. (SBI or SBIs or (screening adj3 “brief intervention*”)).mp.
3. 1 or 2

- DIGITAL TRAINING, /

1. exp Digital Technology/
2. exp Mobile Applications/
3. exp Internet-Based Intervention/
4. exp Computers, Handheld/
5. exp Internet/
6. (digital adj3 (tech* or intervention*)).mp.
7. ((mobile or smart or smartphone or “smart-phone”) adj3 (app or apps or application* or device*)).mp.
8. (computer* adj2 (handheld or tablet*)).mp.
9. (internet or online or on-line or web or app-based or technology-based).mp.
10. (e-health or ehealth).mp.
11. 4 or 5 or 6 or 7 or 8 or 9 or 10 or 11 or 12 or 13
12. 3 and 14

limit 15 to (english language and yr="2001 -Current")

1. medical education.mp. or exp Education, Medical/
2. (healthcare training or health care training).mp.
3. health educat*.mp. or exp Health Education/
4. health promotion.mp. or exp Health Promotion/
5. exp Health Knowledge, Attitudes, Practice/
6. exp Education, Continuing/
7. (elearning or "e-learning").mp.
8. Combine with OR
9. Combine all searches using AND
10. limit 52 to yr="2001 -Current"
11. limit 59 to english language
12. limit 60 to humans
