# Supplementary material for: The effectiveness of digital training on screening, brief interventions, and referral to treatment (SBIRT) for medical and health professionals: a systematic review
Source: Br Med Bull. 2025 Sep 22;156(1):ldaf013. doi: 10.1093/bmb/ldaf013 (PMC12452273; doi:10.1093/bmb/ldaf013)
Supplement: Supplementary_file_S2_ldaf013 [file supplementary_file_s2_ldaf013.docx]

**Supplementary file S2.** Critical appraisal checklists and results tables

Supplementary Table A – JBI Critical appraisal checklist for randomized controlled trials

**JBI Critical Appraisal Checklist for Randomized Controlled Trials**

Reviewer Date

Author Year Record Number

Yes No Unclear NA

1. Was true randomization used for assignment of
participants to treatment groups? □ □ □ □

2. Was allocation to treatment groups concealed? □ □ □ □

3. Were treatment groups similar at the baseline? □ □ □ □

4. Were participants blind to treatment assignment? □ □ □ □

5. Were those delivering treatment blind to
treatment assignment? □ □ □ □

6. Were outcomes assessors blind to treatment
assignment? □ □ □ □

7. Were treatment groups treated identically other
than the intervention of interest? □ □ □ □

8. Was follow up complete and if not, were differences
between groups in terms of their follow up adequately
described and analyzed? □ □ □ □

9. Were participants analyzed in the groups to which they
were randomized? □ □ □ □

10. Were outcomes measured in the same way for
treatment groups? □ □ □ □

11. Were outcomes measured in a reliable way? □ □ □ □

12. Was appropriate statistical analysis used? □ □ □ □

13. Was the trial design appropriate, and any deviations from
the standard RCT design (individual randomization, parallel
groups) accounted for in the conduct and analysis of the trial? □ □ □ □

Overall appraisal:
Include □Exclude □ Seek further info □

Comments Including reason for exclusion)

Supplementary Table B – JBI Critical appraisal checklist for quasi-experimental studies

**JBI Critical Appraisal Checklist for Quasi-Experimental Studies**

**(non-randomized experimental studies)**

Reviewer Date

Author Year Record Number

Yes No Unclear NA

14. Is it clear in the study what is the ‘cause’ and what is the ‘effect’ (i.e. there is no confusion about which variable comes first)? □ □ □ □

15. Were the participants included in any comparisons similar? □ □ □ □

16. Were the participants included in any comparisons receiving similar treatment/care, other than the exposure or intervention of interest? □ □ □ □

17. Was there a control group? □ □ □ □

18. Were there multiple measurements of the outcome both pre and post the intervention/exposure? □ □ □ □

19. Was follow up complete and if not, were differences between groups in terms of their follow up adequately described and analyzed? □ □ □ □

20. Were the outcomes of participants included in any comparisons measured in the same way? □ □ □ □

21. Were outcomes measured in a reliable way? □ □ □ □

22. Was appropriate statistical analysis used? □ □ □ □

Overall appraisal:
Include □Exclude □ Seek further info □

Comments (Including reason for exclusion)

Supplementary Table C JBI Critical Appraisal Checklist Results

|  |  |  | | Question number | | | | | | |  | | | | | | | |
| --- | --- | --- | --- | --- | --- | --- | --- | --- | --- | --- | --- | --- | --- | --- | --- | --- | --- | --- |
|  | **RANK** | **1** | **2** | | **3** | **4** | **5** | **6** | **7** | **8** | | **9** | **10** | **11** | **12** | **13** | **TOTAL** |  |
| RCT Studies | Risk of bias thresholds: RCT: Low 13-12, Moderate 11-9, High ≤8 | | | | | | | | | | | | | | | | |  |
| Albright, 2018 | Mod | 1 | 1 | | 1 | 0 | 0 | 0 | 0 | 1 | | 1 | 1 | 1 | 1 | 1 | 9 |  |
| Curtis, 2022 | Mod | 1 | 0 | | 1 | 0 | 0 | 1 | 1 | 1 | | 1 | 1 | 1 | 1 | 1 | 10 |  |
| Fleming, 2009 | Mod | 1 | 0 | | 1 | 0 | 0 | 1 | 1 | 1 | | 1 | 1 | 1 | 1 | 1 | 10 |  |
| Giudice, 2002 | High | 1 | 0 | | 0 | 1 | 0 | 0 | 0 | 0 | | 1 | 1 | 1 | 1 | 1 | 8 |  |
| O’Brien, 2019 | High | 1 | 0 | | 1 | 0 | 0 | 0 | 0 | 1 | | 1 | 1 | 1 | 1 | 1 | 8 |  |
| Ruzek, 2014 | Low | 1 | 1 | | 1 | 1 | 0 | 1 | 1 | 1 | | 1 | 1 | 1 | 1 | 1 | 12 |  |
| Stoner, 2014 | Mod | 1 | 1 | | 1 | 0 | 0 | 1 | 1 | 1 | | 1 | 1 | 1 | 1 | 1 | 11 |  |
| Truncali, 2011 | Mod | 1 | 0 | | 1 | 0 | 0 | 0 | 1 | 1 | | 1 | 1 | 1 | 1 | 1 | 9 |  |
|  |  |  |  | |  |  |  |  |  |  | |  |  |  |  |  |  |  |

Question Response 1= yes; 0 = no;

|  | Question number | | | | | | | | | |  |
| --- | --- | --- | --- | --- | --- | --- | --- | --- | --- | --- | --- |
|  | **RANK** | **14** | **15** | **16** | **17** | **18** | **19** | **20** | **21** | **22** | **TOTAL** |
| Quasi-Experimental | Risk of bias thresholds: Quasi : Low 8, 9, Moderate 7,6, High <6 | | | | | | | | | | |
| Acquavita, 2021 | High | 1 | 0 | 0 | 0 | 1 | 1 | 1 | 1 | 1 | 6 |
| Bernstein, 2007 | High | 1 | 0 | 0 | 0 | 1 | 1 | 1 | 1 | 1 | 6 |
| Bray, 2009 | High | 1 | 0 | 0 | 0 | 1 | 0 | 1 | 1 | 1 | 5 |
| Broyles, 2013 | Low | 1 | 1 | 1 | 1 | 1 | 1 | 1 | 1 | 1 | 9 |
| Bull, 2021 | Mod | 1 | 0 | 1 | 0 | 1 | 1 | 1 | 1 | 1 | 7 |
| Cambron, 2023 | Low | 1 | 1 | 1 | 1 | 1 | 1 | 1 | 1 | 1 | 9 |
| Cordes, 2022 | Low | 1 | 1 | 1 | 0 | 1 | 1 | 1 | 1 | 1 | 8 |
| Gainey, 2022 | Low | 1 | 1 | 1 | 0 | 1 | 1 | 1 | 1 | 1 | 8 |
| Gonzalez, 2020 | Mod | 1 | 0 | 1 | 0 | 1 | 1 | 1 | 1 | 1 | 7 |
| Gonzalez, 2021 | Low | 1 | 1 | 1 | 0 | 1 | 1 | 1 | 1 | 1 | 8 |
| Habib, 2019 | Low | 1 | 1 | 1 | 1 | 1 | 0 | 1 | 1 | 1 | 8 |
| Kelly, 2018 | Low | 1 | 1 | 1 | 1 | 1 | 0 | 1 | 1 | 1 | 8 |
| Knopf-Amelung, 2018 | Low | 1 | 1 | 1 | 1 | 1 | 0 | 1 | 1 | 1 | 8 |
| Lee, 2008 | Low | 1 | 1 | 1 | 1 | 1 | 0 | 1 | 1 | 1 | 8 |
| Martin, 2020 | Low | 1 | 1 | 1 | 1 | 1 | 1 | 1 | 1 | 1 | 9 |
| Mitchell, 2017 | High | 1 | 0 | 0 | 0 | 1 | 1 | 1 | 1 | 1 | 6 |
| Osborne, 2016 | High | 1 | 0 | 0 | 0 | 1 | 1 | 1 | 1 | 1 | 6 |
| Oster, 2022 | High | 1 | 0 | 0 | 0 | 1 | 0 | 1 | 1 | 1 | 5 |
| Petrides, 2024 | High | 1 | 0 | 0 | 0 | 0 | 0 | 1 | 0 | 1 | 3 |
| Pickard, 2024 | High | 1 | 0 | 0 | 0 | 1 | 0 | 1 | 1 | 1 | 5 |
| Pringle, 2017 | High | 1 | 0 | 0 | 0 | 1 | 0 | 1 | 1 | 1 | 5 |
| Puskar, 2016 | High | 1 | 0 | 0 | 0 | 1 | 0 | 1 | 1 | 1 | 5 |
| Puskar, 2016 | Mod | 1 | 0 | 1 | 1 | 1 | 0 | 1 | 1 | 1 | 7 |
| Puskar, 2016 | High | 1 | 0 | 0 | 0 | 1 | 1 | 1 | 1 | 1 | 6 |
| Putney, 2019 | High | 1 | 0 | 0 | 0 | 1 | 0 | 1 | 1 | 1 | 5 |
| Putney, 2021 | High | 1 | 0 | 0 | 0 | 1 | 1 | 1 | 1 | 1 | 6 |
| Rawlings, 2019 | High | 1 | 0 | 0 | 0 | 1 | 0 | 1 | 1 | 1 | 5 |
| Rittle, 2019 | High | 1 | 0 | 0 | 0 | 0 | 0 | 0 | 0 | 0 | 1 |
| Sanford, 2023 | High | 1 | 0 | 0 | 0 | 1 | 0 | 0 | 1 | 1 | 4 |
| Stevens, 2024 | High | 1 | 0 | 0 | 0 | 0 | 0 | 0 | 0 | 0 | 1 |
| Tanner, 2012 | Low | 1 | 1 | 1 | 0 | 1 | 1 | 1 | 1 | 1 | 8 |
| Tenkku Lepper, 2019 | High | 1 | 0 | 0 | 0 | 1 | 1 | 0 | 1 | 1 | 5 |
| Wacker, 2023 | Mod | 1 | 0 | 0 | 1 | 1 | 1 | 1 | 1 | 1 | 7 |
| Wood, 2022 | Low | 1 | 1 | 1 | 0 | 1 | 1 | 1 | 1 | 1 | 8 |
| Question Response 1= yes; 0 = no; | | |  |  |  |  |  |  |  |  |  |

Supplementary Table D JBI Critical Appraisal Checklist Results

Table E Data Extraction Form

| **Study ID** |  | Country |  |
| --- | --- | --- | --- |
| Lead Author |  | Publication Year |  |
| Title |  | Design |  |
| Blinding | Y/N | Dates |  |
| Conflict of interests |  | Funding |  |
| Aims |  | Setting/ Context |  |
| Participant Job Role |  | No of Participant |  |
| Control |  |  |  |
| Digital element |  |  |  |
| Inclusion |  |  |  |
| Exclusion |  |  |  |
| Mean age of participants |  | Female %  Ethnicity: White % |  |
| **Outcomes** |  |  |  |
| Pretest Control |  | Pretest-Intervention |  |
| Post-test control |  | Post-test Intervention |  |
| **NOTES** |  |  |  |
|  |  |  |  |
